# Non-Steroidal Anti-Inflammatory Drugs and Associated Toxicities in Horses

**DOI:** 10.3390/ani12212939

**Published:** 2022-10-26

**Authors:** Jordan Flood, Allison J. Stewart

**Affiliations:** School of Veterinary Science, University of Queensland Gatton, 5391 Warrego Highway, Gatton, QLD 4343, Australia

**Keywords:** colic, colitis, analgesia, right dorsal colitis, renal papillary necrosis, equine gastric ulcer syndrome

## Abstract

**Simple Summary:**

Non-steroidal anti-inflammatory drugs (NSAIDs) are regularly used in equine veterinary medicine for their analgesic and anti-inflammatories properties. NSAIDs have a reportedly narrow margin of safety with associated toxicities in the horse including renal papillary necrosis, equine gastric ulcer syndrome and right dorsal colitis. This review collates current literature on the use of NSAIDs in horses and its associated toxicities.

**Abstract:**

Effective pain management in horses can be a challenge despite the understanding that appropriate analgesia improves animal welfare and increases treatment success. The administration of NSAID drugs, particularly phenylbutazone and flunixin, are common practice in equine veterinary patients. Known for their analgesic and anti-inflammatory properties, NSAIDs are used for the treatment of a variety of conditions in horses, from gastrointestinal to orthopedic pain. Despite extensive usage, NSAIDs have a narrow margin of safety and the body of literature documenting the efficacy and side effects of different NSAIDs is broad. The three main side effects associated with excessive or prolonged NSAID usage in horses include gastroduodenal ulceration, right dorsal colitis (RDC) and renal papillary necrosis. The use of cyclooxygenase-2 selective NSAIDS, such as firocoxib, are theoretically safer. The aim of this paper is to review the current literature on the use and efficacy of different NSAIDs, summarise the associated side effects of NSAID usage and evaluate the current state of knowledge for the diagnosis and treatment of such toxicities.

## 1. Introduction

Pain management is an integral component of human and veterinary medicine. The International Association for the Study of Pain (IASP) defines pain as ‘an unpleasant sensory and emotional experience associated with, or resembling that associated with, actual or potential tissue damage’ [1] (p. 1976). Adequate analgesia is essential for minimising physiologic stress, improving animal welfare, convalescence and increasing treatment success [2,3]. Non-steroidal anti-inflammatory drugs (NSAIDs) are one of the most commonly prescribed classes of drugs for analgesia in human and veterinary medicine. In addition to their analgesic effects, NSAIDs are renowned for their anti-inflammatory and anti-pyretic properties [4].NSAIDs are frequently used in equine veterinary practice to treat a variety of conditions, including gastrointestinal pain (colic), muscular and orthopaedic pain, peri-operative pain, corneal ulcers, uveitis and laminitis [5].

Despite widespread use in equine medicine, NSAIDs have a narrow margin of safety with potentially adverse and fatal side effects [6,7]. Several toxicities have been associated with this class of drug in both humans and animals [6]. Individual sensitivity to NSAID toxicity exists, with potential side effects able to occur even when recommended dose ranges are followed. Adverse events from NSAID toxicity can be fatal, therefore, it is important clinicians can recognise early symptoms and diagnostic procedures for identification of NSAID toxicosis and adopt a multi-modal approach to analgesia where possible. The three main side-effects associated with NSAID use in horses include right dorsal colitis (RDC), gastroduodenal ulceration and renal papillary necrosis [6,7]. The purpose of this review is to collate existing literature of usage of NSAIDs and their associated toxicities in equine medicine and summarise findings into one readily-accessible document.

## 2. Methodology

Resources included in this review article were English based articles and textbooks sourced from a literature search through Web of Science, Pubmed and the University of Queensland library databases. Resources were included if they contained original data or information on NSAID use or toxicities in horses. Studies on NSAIDs, NSAID usage or NSAID related toxicities in humans or other species were included if the information was potentially relevant to equine medicine. Literature searches in the various databases were based upon the following keywords: NSAID use in horses, NSAID toxicities in horses, right dorsal colitis in horses, renal papillary necrosis in horses, COX-2 selective NSAIDs and NSAID toxicity. The date of the last literature search performed was on 6 October 2022.

## 3. Pathophysiology of NSAID Mechanism of Action

**Figure 1:**
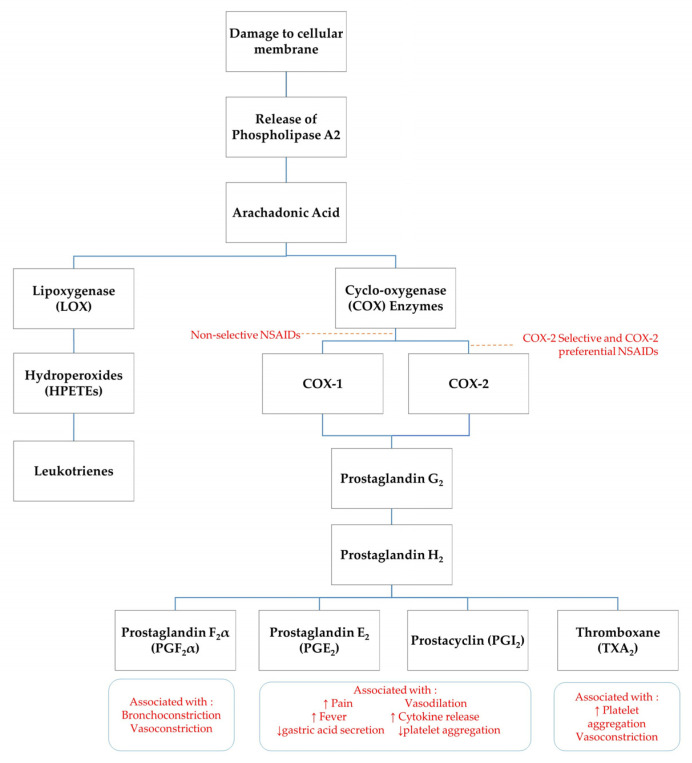
Schematic of arachidonic acid cascade. ↑ indicates increase, ↓ indicates decrease.

NSAIDs exert their analgesic effects through peripheral and central modalities. Cellular damage results in stimulation of specific cell-surface receptors which activate phospholipase A_2_ to release the short chain fatty acid, arachidonic acid, and a pro-inflammatory, platelet-aggregation factor from the cell membrane [8,9]. The production of arachidonic acid initiates the process termed ‘the arachidonic cascade’ [5]. Arachidonic acid acts as a substrate for a number of enzymes including cyclo-oxygenase (COX), 5-lipoxygenase (5-LO), 12-lipoxygenase (12-LO) and 15-lipoxygenase (15-LO). COX enzymes are responsible for the production of several pro-inflammatory eicosanoids such as prostacyclins, prostaglandins and thromboxane (TXA_2_) [9]. An unstable form of prostaglandins, prostaglandin G_2_, is produced by oxygenation of arachidonic acid via COX enzymes. Prostaglandin G_2_ is converted into prostaglandin H_2_, which is further converted to other specific prostaglandins such as TXA_2_, prostaglandin F_2_α (PGF_2_α) and prostaglandin E_2_ (PGE_2_) (see Figure 1) [5]. Prostanoids do not directly activate nociceptors, however they can trigger hyperalgesia by sensitising nociceptors to mechanical stimuli and chemical mediators, such as bradykinin and histamine [5,9]. Other prostanoid pro-inflammatory mechanisms include dilation of arterioles resulting in an increased blood flow to the site of injury [8].

The primary mode of action of NSAIDs is via inhibition of COX enzymes, thereby limiting prostaglandin synthesis. Some studies suggest NSAIDs also have a centrally mediated mechanism of action, with analgesic activity in sheep reversed by administration of opioid and α_2_-adrenergic receptor inhibitors [10]. Further investigation into central mechanisms of NSAID analgesic activity is required. There are two main COX enzyme isoforms recognised in horses: COX-1 and COX-2. A third COX enzyme, COX-3, has found to be a constitutively expressed in canine and rodent models, however no research to establish the significance of COX-3 enzymes in horses has been published [11,12,13]. COX-1 is a constitutively expressed enzyme found in nearly all healthy tissues. It is responsible for maintaining physiologic homeostasis in many organ systems and the synthesis of PGE_2,_ prostaglandin I_2_ and TXA_2_, which have cytoprotective effects on gastric function and secretion and platelet aggregation [9] COX-1 is found in abundance in the gastrointestinal tract, kidneys and platelets, hence inhibition of prostaglandins through NSAID administration can have adverse effects on these cells [14].

The COX-2 isoform is considered to be an inducible enzyme upregulated in the presence of inflammatory stimuli, including inflammatory cytokines and bacterial lipopolysaccharides [5]. Previously COX-2 has been associated principally with pain and inflammation, however recent findings show that COX-2 isoforms are constitutively expressed in tissues including kidneys, nervous system (brain and spinal cord), reproductive tract (ovary, uterus, placenta), thymus, bone, cartilage, synovia, endothelia, prostate, and lung [14]. COX-2 has important roles in cellular processes such as apoptosis, gene expression and differentiation, bone remodelling, wound healing and neoplasia [14]. In humans and other species, prostaglandins which have a protective role in the gastrointestinal mucosa are derived from not only COX-1, but also COX-2 [15].

## 4. Comparison of NSAIDs

Different NSAIDs have varying efficacies, uses and dosages despite similar fundamental mechanisms of action. Non-selective NSAIDs, such as phenylbutazone and flunixin, inhibit both COX-1 and COX-2 enzymes and are theoretically and anecdotally more likely to have higher rates of toxic side effects. Newly formulated NSAIDs, such as meloxicam and firocoxib, have been developed for targeted, selective inhibition of COX-2 to reduce potential side effects from inhibition of the homeostatic-protective prostaglandins derived from COX-1. In vitro assays are used to determine the inhibitory concentrations (IC_50_) for COX 1 and COX 2 enzymes, thus determining whether an NSAID is non-selective or COX-2 selective [5]. The ratio of COX 1: COX 2 selectivity for the same drug varies amongst different species, and there is also likely individual variation. Common NSAIDs used in equine practice and their relative route, recommended dose and COX-1: COX-2 selectivity for horses is listed in Table 1 for adults and Table 2 for foals below.

Phenylbutazone is the most commonly administered NSAID in equine medicine. It is often prescribed for orthopaedic pain whereas flunixin meglumine is the preferred analgesic for colic and visceral pain, however, there is little evidence to support such tissue selectivity [5,7,18,27]. In horses, the administration of flunixin has been found to delay repair of the small intestinal mucosa, increase neutrophil infiltration into the intestinal mucosa and increase permeability to lipopolysaccharides after ischaemic injury [28]. The same study found that meloxicam had an equal or greater analgesic effect without the side effects of flunixin, suggesting meloxicam may be preferred for treatment of small-intestinal related colic [28]. While some studies have investigated the pharmacodynamics and safety of meloxicam in horses, further research is required to identify the clinical efficacy of meloxicam administration for colic [29]. A randomised, double-blinded, placebo controlled clinical field study found that the administration of meloxicam (0.6 mg/kg IV q24 h) was effective at controlling pain post orthopaedic surgery [30]. Meloxicam has also been found to reduce lameness, effusion and joint inflammation associated with acute synovitis and reduce inflammation-mediated cartilage catabolism in vivo [31]. There is no published data comparing the analgesic efficacy of phenylbutazone to meloxicam for orthopaedic pain.

It is well documented that phenylbutazone provides effective analgesia for orthopaedic pain in horses [5,32,33]. The recommended dose of phenylbutazone is 4.4 mg/kg every 12 h on the first day of administration, followed by a reduced dose of 2.2 mg/kg every 12 h on consecutive days of use [7]. Maintaining a dose rate of 8.8 mg/kg/day compared to 4.4 mg/kg/day provided no additional analgesia to clinically lame horses, therefore exceeding the recommended dose only increases the risk of toxicity with no analgesic benefit. Phenylbutazone and flunixin have been found to provide comparable levels of analgesia for horses with navicular syndrome treated at clinical doses for 4 days [34]. Several studies have evaluated the effectiveness of phenylbutazone compared to firocoxib and ketoprofen. In experimentally induced synovitis, a single dose of phenylbutazone (4.4 mg/kg) was found to be more effective at reducing lameness, synovial fluid volume and joint temperature in comparison to ketoprofen (2.2–3.6 mg/kg) [35].Firocoxib (0.1 mg/kg PO q24 h) was found to be comparable to phenylbutazone (4.4 mg/kg PO q24 h) for analgesia in naturally occurring osteoarthritis in horses. Firocoxib was found to provide significantly improved pain scores after manipulation or palpation, joint circumference, and range of motion scores after two weeks of administration, however phenylbutazone was superior for overall lameness reduction and scoring of joint swelling [36]. A randomised, controlled trial with administration of firocoxib (0.1 mg/kg) for 14 days to horses with osteoarthritis found that improvement in lameness was detected in 80% of horses by the end of the trial [37]. Similar results have been replicated in canine studies of osteoarthritis, with firocoxib administration improving lameness scores at a walk and trot, pain on palpation and range of motion in comparison to etodolac [38]. Current publications suggest that phenylbutazone appears to be the most effective analgesic for orthopaedic pain in comparison to COX-2 selective NSAIDs, however it is difficult to compare studies when different dosages are used.

NSAIDs are frequently administered to ameliorate clinical signs of inflammation, poor tissue perfusion and sepsis associated with colic, colitis, and abortions. It is hypothesised that arachidonic metabolites, including PGF2α, thromboxane A2 and PGE_2_, are involved in abdominal pain, diarrhoea and clinical signs associated with toxic shock, therefore the COX inhibitory action of NSAIDs can reduce the complications of uncontrolled inflammation [39,40]. Several studies in the 1980’s evaluated the use of flunixin meglumine after experimental endotoxin administration to horses. Pre-treatment of ponies with flunixin (1.1 mg/kg IV) prior to administration of *Escherichia coli* O55:B5 endotoxins prevented the development of lactic acidosis, arterial hypoxemia, diarrhoea, colic, ataxia and respiratory difficulties that were observed in ponies administered endotoxin alone [39]. A low-dose of flunixin meglumine (0.25 mg/kg) suppressed blood lactate, plasma eicosanoids, thromboxane B2 and 6-ketoprostaglandin F10, in horses that had endotoxin administered experimentally. The lower dose of flunixin (0.25 mg/kg) also reduced clinical symptoms of sepsis including abdominal discomfort, tachypnoea and hyperthermia [39,41]. This has led to the commonly used ‘anti-endotoxic dose’ of flunixin meglumine (0.25 mg/kg) in equine practice. It is important to realise that it is the COX suppression of the arachidonic acid cascade that is sufficient to reduce the effects of endotoxin administration and that flunixin itself does not bind endotoxins, as has been reported for drugs such as polymyxin B [42].

Flunixin meglumine has been shown to impair epithelial restitution and compromise small intestinal barrier function, with increases in permeability of lipopolysaccharides after ischaemic injury [28,43]. The deleterious effect of flunixin meglumine on the epithelial barrier was inhibited by post-treatment administration of the PGE_1_ analogue, misoprostol [43]. When compared to flunixin meglumine (1.1 mg/kg IV), the COX-2 preferential NSAID, meloxicam (0.6 mg/kg IV) did not inhibit the mucosal repair of the ischaemic mucosa, with similar findings to a placebo saline solution [44]. In addition, the post-operative pain scores, heart rate and respiratory rate for meloxicam were comparable or greater than the effect of flunixin meglumine [44]. The clearance of meloxicam was slower in this study in comparison to previously reported values, therefore further investigation into drug dose and frequency of meloxicam administration in critically ill horses is recommended [44]. Investigations into the use of oral meloxicam have also been performed, with pre-treatment oforal meloxicam (0.6 mg/kg) found to reduce pain scores in thoroughbreds administered a low-dose lipopolysaccharide challenge [45].

The use of COX-2 selective NSAIDs in equine colic and sepsis is increasing in popularity. A multicentre, blinded, randomised clinical trial comparing administration of flunixin meglumine (1.1 mg/kg IV q12 h) to firocoxib (0.3 mg/kg loading dose, 0.1 mg/kg IV q24 h) found that there was no difference in pain scores post operatively between the two NSAID groups, indicating similar levels of analgesia [46]. The flunixin-treated group had more horses with increased sCD14, a serum marker of endotoxaemia, compared to those administered firocoxib. The firocoxib-treated horses had reduced small intestinal permeability to lipopolysaccharides after an 18 h recovery period in comparison to horses treated with flunixin meglumine [46]. However, the flunixin-treated group contained 1.5 times the number of horses requiring resection and anastomosis than the firocoxib group, which may affect the recovery rates and hence the clinical relevance of this study [47]. Concerns have been raised regarding the use of COX-2 selective NSAIDs, such as firocoxib, in equine colic cases, as recent developments in human medicine have shown that COX-2 enzymes are essential for colorectal healing, and the use of COX-2 inhibitory drugs may impair anastomotic healing in human patients [47,48]. Further research is required to determine the clinical relevance of COX-2 on anastomotic healing in the equine gastrointestinal system.

Overall, current literature would suggest that COX-2 selective drugs are seemingly safer than non-selective NSAIDs in horses, with fewer published case reports and prospective studies of associated toxicities. Meloxicam and firocoxib are currently more commonly used and researched COX-2 preferential and COX-2 selective drugs in horses [49,50]. Studies suggest these COX-2 selective NSAIDs provide comparable levels of analgesia to non-selective NSAIDs for conditions such as small-intestinal colic and lameness, with fewer complications, particularly with large doses or chronic use [36,37,49,51]. Further research, ideally randomised studies with larger sample sizes, is required to determine the analgesic effect of COX-2 selective NSAIDs for conditions in equine veterinary medicine and their pharmacokinetics in the critically ill horses [50]. Other newer COX-2 selective drugs, such as vitacoxib and celecoxib, have recently had pharmacokinetic studies performed in horses [23,52]. While no analgesic studies have yet been published, these drugs may provide future avenues for analgesia in equine veterinary medicine.

## 5. Predisposing Factors Leading to Toxicity

NSAIDs have a narrow therapeutic range and although relatively safe at recommended doses, individual sensitivity may predispose some horses to toxicosis [4]. Conditions that reduce tissue perfusion and drug elimination, such as renal disease, dehydration and hepatic disease, may predispose horses to NSAID toxicosis. Sepsis may secondarily cause reduced tissue perfusion and hypovolemia, thus predisposing horses to NSAID toxicity. Concurrent administration of more than one different type of NSAID is known as ‘stacking’ [53]. This practice is contraindicated, with NSAIDs producing an additive effect so that two NSAIDs co-administered at recommended doses is equivalent to administering double the recommended dose of one NSAID. For example, concurrent administration of the recommended dose of phenylbutazone and flunixin (phenylbutazone 2.2 mg/kg, PO q12 h; flunixin meglumine, 1.1 mg/kg, IV q12 h) had a significant decrease in plasma protein levels and increased incidence of gastric ulceration after 5 days of treatment in comparison to a placebo-controlled group and individual phenylbutazone administration (2.2 mg/kg PO q12 h) alone [54]. Concurrent administration of phenylbutazone and firocoxib for 10 days resulted in elevated creatinine concentrations, decreased total protein concentrations and reductions in urine specific gravity, consistent with protein losing enteropathy and renal toxicosis [55]. Administration of an NSAID with a nephrotoxic drug, such as an aminoglycoside, oxytetracycline or polymyxin B, may also increase the risk of nephrotoxicity in horses [14]. Ponies are also reported to have an increased susceptibility to NSAID toxicity [56,57].

Foals may be more predisposed to NSAID toxicity in comparison to adult horses due to an increased volume of body fluid, altered activities of drug-metabolising enzymes, such as increased activity of cytochrome P450 when liver volume is normalised to body weight, and often lower plasma protein concentrations in comparison to adults [25]. Neonatal foals are reported to have significantly lower clearance and longer half-life of non-selective NSAIDs such as flunixin meglumine, ketoprofen and phenylbutazone in comparison to older foals and adult horses [24,58,59]. Healthy neonatal foals aged 48–72 h old administered the recommended dose of flunixin meglumine (1.1 mg/kg IV q24 h) for five days did not display any clinical or pathological abnormalities, however foals that received six times the recommended flunixin meglumine dose (6.6 mg/kg IV q24 h) developed gastric and intestinal ulceration and cecal petechiation [60]. In another study, foals administered the label dose of flunixin (1.1 mg/kg group-1 PO, group-2 IV) for 30 days developed erosions in the glandular gastric portion of the stomach [61]. Pharmacokinetic studies of meloxicam (0.6 mg/kg PO q12 h) and firocoxib (0.1 mg/kg PO q24 h) in neonatal foals report an increased clearance and reduced half-life of these drugs in neonatal foals compared to adult horses [19,62]. These findings suggest that a reduced dosage interval or increased dose rate of COX-2 selective drugs may be required to reach therapeutic concentration in foals. Further research is required to determine appropriate therapeutic dosages and safety margins of COX-2 selective NSAIDs in both healthy and compromised foals.

## 6. Pathology

### 6.1. Equine Gastric Ulcer Syndrome (EGUS) and Equine Glandular Gastric Disease (EGGD)

The administration of NSAIDs and its contribution to the development of EGUS and EGGD is controversial [63,64]. While there is evidence that gastric glandular ulcers develop after administration of NSAIDs above recommended dosages, there is limited evidence to suggest NSAID usage contributes to EGUS at therapeutic dosages [64]. The pathogenesis of NSAIDs in the development of EGUS is believed to be multifactorial, with the inhibition of COX enzymes impacting several mechanisms for gastric protection and healing. Inhibition of COX enzymes leads to increased gastric acid secretion and therefore a decrease in pH and output of bicarbonate and mucous. Inhibition of COX enzymes also impairs vasodilation and gastric blood flow [4,56]. Prostaglandins, particularly PGE_2_ and PGI_2,_ are crucial for mucosal health and repair including epithelial restitution and mucosal cell turnover [4,56,65]. These prostaglandins have significant roles in maintaining epithelial tight junction integrity, which is an integral mechanism for mucosal repair after injury and mucosal barrier function [56,66].

The most commonly reported NSAID contributing to gastrointestinal damage is phenylbutazone, likely due to its non-selective COX inhibition and therefore lower safety profile [27,56]. Administration of 4.4 mg/kg PO q12 h for 7 days induced equine glandular gastric disease graded at ≥2 in all horses treated, with pathology most severely affecting the pylorus or pyloric antrum [63]. Despite an increase in EGGD, there was no significant difference observed between phenylbutazone-treated and placebo-control group for presence of squamous gastric ulcers [63]. In another study, horses administered 4.4 mg/kg PO q24 h of phenylbutazone for five days with a single overdose of 13.2 mg/kg PO on day 6 were found to have increased pathology in the glandular gastric mucosa, decreased anti-oxidants (nitrous oxide, superoxide dismutase and catalase) and increased oxidant (malondialdehyde) parameters in comparison to placebo-saline control horses [67]. This indicates that oxidative stress may be a potential mechanism of gastric injury after phenylbutazone administration [67]. Phenylbutazone (4.4 mg/kg IV q8 h) was also reported to most severely affect the glandular portion of the stomach in horses in comparison to flunixin meglumine (1.1 mg/kg IV q8 h) and ketoprofen (2.2 mg/kg IV q8 h) when administered for a duration of 12 days [68]. The total doses administered in this study far exceed the manufacturer’s recommendations and may not be a true reflection of pathology when administered at recommended doses. Gastric mucosal lesions at necropsy 48 h after a single overdose of phenylbutazone (13.46 mg/kg IV) revealed sloughing of gastric surface epithelium, necrosis of lamina propria, microthrombi and degeneration of subsurface capillaries, with the endothelium ranging from swollen to lysed and necrotic [69]. This suggests phenylbutazone may also cause microvascular injury of the gastric mucosa.

COX-1 enzymes have been found in healthy, non-ulcerated, squamous gastric mucosa in horses, with little expression of COX-2 isoforms [70]. However, in the presence of ulcerated lesions, it was found that expression of COX-2 enzymes was significantly increased. COX-2 enzymes may have an important role in the mucosal healing of squamous gastric ulcerations in horses and further research is required to assess the clinical relevance and potential effects of COX-2 selective NSAIDs on the healing of gastric ulcers [70]. A study comparing meloxicam (0.6–3.0 mg/kg PO) and phenylbutazone (4.4 mg/kg PO q12 h day 1, 2.2 mg/kg PO q12 h for 4 days, 2.2 mg/kg PO q24 h for 9 days) found that phenylbutazone administration resulted in increased gastric mucosal permeability to sucrose, suggesting phenylbutazone may be associated with increased mucosal damage in comparison to meloxicam [71]. Similar results were reported in another study comparing phenylbutazone (4.4 mg/kg PO q24 h) and firocoxib (0.1 mg/kg PO q24 h) administration for 10 days [51]. Gastroscopy and faecal myeloperoxidase, a non-invasive marker of lower gastrointestinal inflammation, were measured in both NSAID groups and a placebo-control group. Both firocoxib and phenylbutazone treated horses had increased squamous gastric ulcers in comparison to the placebo-control group after 10 days of treatment [51]. Glandular gastric lesions were significantly increased in both NSAID treated groups, however lesions were more severe in horses treated with phenylbutazone. Faecal myeloperoxidase remained unchanged between day 0 and 10 in the placebo-control and firocoxib groups, although concentrations were elevated in horses treated with phenylbutazone [51]. While there are few publications comparing non-selective and COX-2 selective NSAID induced gastric ulceration, it appears that COX-2 selective drugs result in less damage to the gastric mucosa.

Proton pump inhibitors (PPI) are the most common class of drugs used to treat squamous gastric ulceration. Historically PPI’s have been administered prophylactically with concurrent NSAID administration to reduce the gastrointestinal side effects associated with NSAIDs [72]. Recent evidence in human medicine raised concerns over the safety of co-administering PPI’s and NSAID’s, with reports that NSAID enteropathy may be worsened with concurrent administration [73,74]. Experimental administration of omeprazole (10 mg/kg IP q12 h) and naproxen (10 mg/kg PO q12 h) in rats found that intestinal damage was significantly worse in rats receiving both PPI and NSAID in comparison to rats receiving NSAID or PPI alone [72]. In the NSAID-only treated group, there were low levels of haemorrhagic damage in the gastric mucosa and intestine. In the co-administered PPI and NSAID group, gastric damage was not observed however blood and ulcerations were evident in the lumen of the intestines. A significant decrease in haematocrit was also seen in the co-administered PPI and NSAID group that was not seen in either NSAID-only or PPI-only group [72]. It is hypothesised that dysbiosis in the small intestine has a role in the pathogenesis of exacerbated gastrointestinal damage with co-administration of PPI and NSAIDs [72]. This hypothesis is further supported in a study which analysed the gastrointestinal permeability in dogs receiving a combination of omeprazole (1 mg/kg PO q12 h) and carprofen (4 mg/kg PO q24 h) in comparison to a placebo and carprofen alone [75]. It was found that the combination of carprofen and omeprazole increased intestinal inflammatory markers and induced faecal dysbiosis in healthy dogs [75]. As concurrent NSAID administration and omeprazole prophylaxis is common in equine veterinary medicine, further analysis of faecal microbiota and dysbiosis associated with this practice is warranted based on results from other species.

A recent study investigated the concurrent use of phenylbutazone and omeprazole in horses [76]. Horses were divided into three treatment groups; phenylbutazone (4.4 mg/kg PO q12 h), phenylbutazone plus omeprazole or placebo-control. Horses were treated for up to 14 days. Glandular gastric ulceration scores increased in the phenylbutazone only group, however no increase in scores were observed in the phenylbutazone/omeprazole group, suggesting that omeprazole may prevent the development of NSAID-induced gastric ulceration [76]. There was increased incidence of intestinal adverse events in both phenylbutazone and phenylbutazone/omeprazole groups, with 25% and 75%, respectively, of horses in each group suffering from complications [76]. Intestinal adverse reactions included colic, impactions, diarrhoea, enterocolitis and typhlocolitis resulting in the euthanasia of 2 horses. It is unknown whether these complications were a result of alterations in intestinal motility, exacerbation of NSAID-induced dysbiosis or intestinal inflammation and/or ulceration [76]. Although the dosage of phenylbutazone was double the recommended dosage, these findings are particularly concerning and highlights the importance for further research into concurrent administration of NSAIDs and PPIs in horses.

Sucralfate is another common class of drug co-administered with NSAIDs for its gastroprotective effects. Sucralfate is thought to have multiple mechanisms of action, including mucosal synthesis, formation of a protective mucosal barrier, buffering of hydrogen ions and encouraging luminal secretion of PGE_2_ leading to increased epithelial cell restitution [77]. Pharmacokinetic studies investigating the drug interaction between sucralfate and NSAIDs in humans suggest that co-administration of the drugs may lead to delayed absorption of NSAIDs, however no studies investigating drug interaction on pharmacokinetic parameters in horses have been published [77]. One study investigated the use of sucralfate (4 g PO q24 h) or ranitidine (2 mg/kg IV q24 h) co-administered with phenylbutazone (5 mg/kg IV q24 h) in foals for ten days [78]. Sucralfate was found to provide partial protection against the deleterious effects of phenylbutazone with no foal from the sucralfate treated group developing diarrhoea compared to 5/7 and 2/7 foals in the phenylbutazone-only and ranitidine/phenylbutazone groups, respectively, developing diarrhea [78]. The severity of gastric ulcerations was found to be less with co-administration of sucralfate than with the ranitidine/phenylbutazone and phenylbutazone-only treatment groups [78]. Another study compared the effects of sucralfate (20 mg/kg PO q8 h) and omeprazole (1 mg/kg PO q24 h) on adult horses fasted and receiving flunixin (1.1 mg/kg q12 h) for five consecutive days [79]. While equine squamous gastric ulcer disease developed in both treatment groups, overall the gastric scores were lower in the omeprazole treated group, suggesting that omeprazole is superior to sucralfate in reducing the severity of NSAID induced squamous gastric ulceration [79]. Horses in the sucralfate treated group also had a statistically significant increase in right dorsal colon wall thickness at the end of the NSAID treatment period, while the right dorsal colon thickness in the omeprazole treated group remained unchanged [79]. In this study, no horses were reported to experience adverse intestinal events in the omeprazole and flunixin treated group [79]. Further research on the drug interactions between sucralfate and omeprazole co-administered with NSAIDs in horses is required.

### 6.2. Renal Papillary Necrosis

Prostaglandins, particularly PGE_2_ and PGI_2_ are important mediators of electrolyte balance, renal blood flow, and water excretion by the kidneys [4,5]. COX-1 and COX-2 enzymes are constitutively expressed in the kidneys and are responsible for the production of these prostaglandins. Inhibition of these prostaglandins through NSAID administration can affect the autoregulatory response to hypoperfusion of the kidney, thus hypovolemia, dehydration and renal disease will predispose horses to renal-induced NSAID toxicity. In a well hydrated horse, it is unlikely that NSAID administration will have detrimental effects to the kidneys, however in a dehydrated horse, reduced prostaglandin production results in vasoconstriction of the afferent arterioles [5]. This results in redistribution of blood flow to the renal cortex and loss of medullary perfusion, resulting in renal papillary necrosis [4,5]. Renal papillary necrosis may be associated with nephrolithiasis, ureterolithiasis or chronic renal failure [4].

Phenylbutazone is the NSAID most commonly associated with renal papillary necrosis in horses, humans and rats [80]. Well demarcated focal medullary necrosis and secondary renal cortical lesions have been reported in horses receiving phenylbutazone (ranging from 0.5 g to 4 g PO q12 h) [80]. In an experimental study, all five horses in a treatment group receiving phenylbutazone (8.8 mg/kg/day PO for 4–90 days) and then deprived of water for 36 to 48 h prior to euthanasia developed acute renal papillary necrosis [81]. Groups receiving phenylbutazone and no water deprivation, and water deprivation only did not develop acute kidney injury [81]. While the amount of phenylbutazone administered in this study greatly exceeds recommended dosage rates and duration of treatment, individual sensitivity to toxicity is likely, and would be exacerbated by concurrent disease such as sepsis, pigmenturia or administration of other drugs associated with nephrotoxicity. Further research is warranted to determine the maximum drug dose or time of water deprivation before even mild acute renal papillary necrosis occurs in horses administered phenylbutazone or other NSAIDs.

### 6.3. Right Dorsal Colitis

Right dorsal colitis (RDC) is an NSAID-induced protein losing enteropathy characterised by marked oedema and thickening localised to the right dorsal colonic walls with focal, linear, or extensive ulcerative lesions into the mucosa and lamina propria [6,82]. It has previously been associated with a high fatality rate, although prognosis has recently been determined to be more favourable, perhaps due to earlier detection and treatment [6,83]. RDC is most commonly associated with excessive and/or prolonged phenylbutazone administration, however cases attributed to the use of other NSAIDs including flunixin meglumine, firocoxib and meloxicam have been reported [6,53]. The pathophysiology of RDC and why lesions are predominantly located in the right dorsal colon is largely unknown. The right dorsal colon is the shortest and widest component of the ascending colon and is a major site of fluid absorption and secretion [28]. It is hypothesised that the inhibition of COX enzymes and subsequent decrease in PGE_2_ and PGF_2_α may compromise the mucosal barrier function, integrity and microcirculation of the right dorsal colon [83]. An in vitro study reported that the right dorsal colon was more susceptible to ischaemia than the small intestine and pelvic flexure. The right dorsal colon was found to have a decreased transepithelial resistance after one hour of ischaemia, with no changes reported in other gastrointestinal segments [6]. Inflammation, necrosis and ischaemia of the right dorsal colon have been reported as post mortem findings in horses that suffered from RDC [84]. Serum hypoproteinemia is a common early finding as plasma proteins leak from mucosal erosions into the intestinal lumen [83].

Acute clinical signs of RDC include colic, anorexia, lethargy, depression, fever and diarrhoea [56]. Severity of colic signs can be variable from mild to severe and may be associated with feeding. Intermittent colic may be attributable to fibrosis and thickening of the right dorsal colon, subsequently leading to impaction colic. Chronic presentations of RDC may manifest as weight loss, intermittent diarrhoea, anorexia, lethargy and ventral oedema with soft faeces [56]. Depending on the duration and severity, affected horses may also present with clinical signs of sepsis and systemic inflammatory response syndrome.

Haematology, serum biochemistry, urinalysis and peritoneal fluid analysis are recommended diagnostics for horses suspected of having RDC [85]. The most consistent and often earliest clinical manifestation of RDC is hypoalbuminaemia [6]. The extent of hypoalbuminemia is often moderate to severe, caused by gastrointestinal protein loss [85]. Panhypoproteinaemia may be observed in more severe and chronic manifestations of RDC. Hypoproteinaemia has been reported in experimentally induced NSAID toxicities in horses without any clinical symptoms or post mortem lesions detected [85]. This highlights the importance of regularly monitoring of serum total albumin or protein concentrations in horses administered NSAIDs, taking into consideration the hydration status of the horse [53]. Serum total hypocalcaemia due to loss of protein-bound calcium and decreased dietary intake may be observed [85]. Anaemia has been reported in around 25% of RDC cases [6]. Usually mild, the anaemia is likely a result of intra-intestinal blood loss and/or anaemia of chronic inflammation. Neutropenia, azotaemia and other electrolyte abnormalities may also be observed depending on severity and duration of RDC. Faecal occult blood testing in horses suspected of RDC is often insensitive and unable to diagnose RDC specifically as it does not differentiate from other parasitic, infectious or inflammatory causes [4].

Confirming a diagnosis of RDC may prove difficult due to the vague clinical symptoms similar to other differential diagnoses including sand enteropathy, ulceration of other areas of the gastrointestinal tract, hepatopathies and inflammatory bowel disease [85]. Historical use of NSAID at prolonged or excessive dosages helps to confirm the diagnosis. However, RDC has been reported after usage of NSAIDs at recommended dosages. Although inaccurate reporting by owners or trainers is possible, individual sensitivity to NSAID toxicity appears to occur. In conjunction with clinicopathologic testing, ultrasonography is considered a useful tool for diagnosis of RDC. The right dorsal colon is most reliably identified ultrasonographically in the 12th intercostal space, ventral to the ventral margin of the right lung lobe [86]. The mean wall thickness in healthy ponies and miniature horses is 0.27 cm and 0.35 cm for large breed horses [86]. In an experimental study of five horses diagnosed with RDC, the range of thickness of the right dorsal colon on ultrasound was reported between 0.82 cm and 1.57 cm [87]. The mean ratio between the mural thickness of the right dorsal colon in comparison to the mural thickness of the right ventral colon was 2.46 in RDC affected horses, and 1.01 in placebo treated, healthy horses [87]. While the use of ultrasonography contributes to a presumptive diagnosis of RDC, it is thought to lack sensitivity as only a small portion of the right dorsal colon is able to be imaged and relies upon the experience of the ultrasonographer. Further investigation is required to determine the sensitivity and specificity of ultrasonography to diagnose RDC. To confirm a diagnosis of RDC, direct visualisation of the right dorsal colon is required via laparoscopy, celiotomy or necropsy [85]. Despite this, surgery is often avoided due to the financial costs, potential complications, and the ability to manage the condition medically if the condition is not severe [85]. Based on history, clinical signs, clinicopathologic and ultrasonographic findings, a presumptive diagnosis of RDC is often made.

Treatment of RDC can include medical or surgical management, however even with aggressive treatment the prognosis may still be poor in severe cases. It is important to discontinue all NSAID drugs [53]. While pain management in these cases is difficult, the use of any non-selective or COX-2 selective NSAID is not recommended. Alternative therapy for analgesia includes opioids, alpha-2 agonists and lidocaine [6]. Dietary management is an integral component of medical treatment. A low-bulk, complete pelleted concentrate feed for approximately three to six months is recommended [85]. Access to roughage should be restricted or eliminated to reduce mechanical and physiological load in the right dorsal colon and should not be reintroduced until serum albumin concentrations return to normal. Feeding a smaller amount of pelleted feed more frequently (4–6 times per day) is suggested. Psyllium mucilloid has been reported to increase the concentration of short-chain fatty acids in the large intestines and promote colonic healing in other animal species [6,85]. Therefore, the addition of psyllium mucilloid is recommended as it may encourage colonic healing in horses.

Dietary supplementation of horses with corn oil significantly increased PGE_2_ in gastric glandular mucosa [88]. While there are no published studies investigating corn oil on right dorsal colonic mucosa, the supplementation of corn oil in the diet is still recommended for horses with RDC for the possible protective mechanisms on the intestinal mucosa and to increase the caloric content of the diet. Over a period of 2 weeks, up to 2 cups of day of corn oil can be slowly introduced to the diet and mixed with a pelleted feed [6]. The high fat content may become unpalatable and may limit the amount that can be fed.

Administration of a synthetic prostaglandin analogue, such as misoprostal (2–5 μg/kg q6–12 h), has been found to prevent ulceration of the gastrointestinal tract as a result of phenylbutazone overdose, therefore is recommended for treatment of RDC [6,85]. Sucralfate is a cytoprotective agent also commonly administered to horses suffering from RDC [6]. However, its efficacy has not been confirmed. The use of metronidazole in horses with RDC has been adapted from the use of metronidazole in human medicine in patients with NSAID enteropathies. In rat models, metronidazole has been demonstrated to inhibit leukocyte adherence to endothelial cells in mesenteric vessels, and therefore exhibits anti-inflammatory effects. Therefore, metronidazole (10–15 mg/kg q8–12 h) may be used as an adjunctive therapy for of RDC [6,85]. The use of an antimicrobial as an anti-inflammatory agent however is generally discouraged as it goes against the principals of responsible antimicrobial use. In horses with RDC demonstrating signs of hypovolaemia, crystalloid fluids must be administered with care. Often these horses also have concurrent hypoproteinaemia and hypoalbuminaemia, and administration of plasma or synthetic colloids are needed to prevent exacerbation of a hypo-oncotic state and worsening oedema [6,85]. Potential sequelae to RDC includes laminitis, therefore prophylactic cryotherapy is recommended for horses exhibiting signs of sepsis. Surgical intervention is often performed when efforts with aggressive medical treatment prove unsuccessful or when colonic fibrosis and stricture occurs causing chronic colic signs. Surgery can be difficult and usually involves resection of the affected right dorsal colon and anastomosis of the diaphragmatic flexure to the small colon [6,85].

Prognosis is dependent on several factors including early detection, aggressive treatment, and the absence of complications such as laminitis, stricture formation or colonic rupture. Previous literature reported mortality rates of nearly 100%, however with improved techniques for early recognition, diagnosis, and treatment of RDC, current literature suggests mortality rates of around 40% [6]. Detection of subclinical disease by the identification of serum hypoalbuminemia can alert veterinarians to the development of RDC. Weekly monitoring of serum albumin or protein concentrations should be performed on horses receiving NSAIDs [6]. If NSAID therapy is likely to be prolonged or serum protein or albumin concentrations begin to decline, then use of COX-2 selective NSAIDs are recommended. If the serum protein concentrations further decline or there is any ultrasonographic evidence of thickening of the right dorsal colon then all NSAIDs are contraindicated.

## 7. Recommendations

NSAIDs should always be administered at recommended dosage rates, however toxicities may still occur despite recommended usage. Further to this, client education on the use and potential complications of NSAIDs should be encouraged. Clients self-administering horses with NSAIDs without consulting a veterinarian is a common cause of NSAID toxicity. Difficulties may also arise when diagnosing an NSAID-related toxicity, as clients may not disclose the actual amount of drugs that has administered to the horse [42]. Only one NSAID should be administered at one time, as co-administration of NSAIDs (known as ‘stacking’) has an additive effect and may pre-dispose horses to adverse events without any additional analgesic benefit [5].

Due to the theoretical increased safety profile of COX-2 selective NSAIDs, it is recommended to use this class of NSAIDs, particularly when prolonged use of NSAIDs is anticipated, for example in conditions such as osteoarthritis [50]. A multimodal approach to equine analgesia should be adopted where possible in attempt to reduce the total NSAID dose or duration [50,89,90]. Appropriateness of alternative therapies vary depending on the degree of pain, if the horse is hospitalized and the presence of hypoproteinemia which may be suggestive of right dorsal colitis, but can in itself result in increased availability of highly protein bound drugs such as many NSAIDs.

The use of acetaminophen (20 mg/kg q12 h) for treatment of pain in horses is gaining popularity following success in other species [13,91]. Although there are few clinical studies exploring the analgesic capacity and side effects of acetaminophen in horses, it can be administered as an inexpensive oral or intravenous analgesic as sole or adjunctive therapy when NSAIDs are contra-indicated, or used in conjunction with NSAIDs to reduce total dose or duration [91]. For hospitalized horses, intravenous lignocaine as a continuous-rate infusion (CRI) is commonly used as an analgesic perioperatively and post operatively, particularly for small intestinal ileus in colic patients [89]. It can also be used as an analgesic to treat severe pain, such as laminitis, as a sole CRI or in combination with ketamine and morphine [90]. Lignocaine, or longer acting local analgesics such as mepivacaine or bupivacaine, may also be utilized to provide perineural analgesia or intraarticular analgesia in combination with opioids. Although horses are prone to excitement or ileus if opioids are used excessively, they are particularly useful in cases with severe pain and are often combined with alpha-2 adrenoreceptor antagonists [89]. Morphine, hydromorphone, butorphanol or buprenorphine can all be used [89]. Topical preservative free morphine can be used for severe ophthalmic pain [92].

Ongoing monitoring should be performed by a veterinarian for any horse receiving an extended course of NSAIDs. Horses receiving a course of NSAIDs should ideally have serum albumin or protein concentrations measured weekly for early detection of gastro-intestinal adverse effects, such as right dorsal colitis [6,7]. Urine specific gravity readings are useful for early detection of renal tubular disease associated with NSAID usage, however the practicality of being able to collect urine may prove unsuccessful. Alternatively, serum creatinine concentrations may also serve as a marker to monitoring renal function for horses receiving NSAIDs. If any symptoms of an NSAID related toxicity occur, NSAID usage should be discontinued immediately.

## 8. Conclusions

Analgesia is an integral component of equine veterinary medicine that can have detrimental effects on equine welfare and healing. NSAIDs are important analgesic drugs utilized in equine veterinary medicine, however, have been associated with adverse side effects. NSAIDs should always be prescribed by a veterinarian, and an analgesic plan that includes continual monitoring for any side effects should be developed. While COX-2 selective drugs are theoretically safer and have fewer reports of adverse events in the literature, it is still possible for toxicities to develop with any NSAID usage. Overall, NSAIDs are a useful class of drugs in equine veterinary medicine but must be administered with appropriate caution.

## Figures and Tables

**Table 1:** Route, recommended dose, IC_50_ COX 1:Cox 2 selectivity, bioavailability, elimination half life (t½), time to maximum concentration (tmax), volume of distribution (Vd) and clearance of common NSAIDs used in equine practice in adult horses.

Drug	Route	Recommended Dosage for Adult Horses	Selectivity (IC_50_ COX 1:COX 2)	Bioavailability	Elimination Half Life (t½)	Time to Maximum Concentration in Plasma (tmax)	Volume of Distribution (Vd)	Clearance
Phenylbutazone	PO, IV	4.4 mg/kg q12 h 1st day, 2.2 mg/kg q12 h subsequent days	0.302 [16]	69–91% [17]	4–6 h [17]	12–14 h (fed) [17]	0.17 L/kg [17]	16–26 mL/h/kg [17]
Flunixin	IV, PO	1.1 mg/kg q12–24 h Anti-endotoxic dose: 0.25 mg/kg q8 h	0.336 [16]	86% [14]	1–2 h [14]	30 min [14]	0.1–0.3 L/kg [14]	
Carprofen	IV	0.7 mg/kg q24 h	1.996 [16]		21.9 h [18]			
Meloxicam	IV, PO	0.6 mg/kg q24 h	3.806 [16]	98% [16]	5.2–8.5 h [16]	1.5 h [16]	0.12 L/kg [16]	34 mL/kg/h [16]
Firocoxib	IV, PO	0.1 mg/kg q24 h	268–643 [19]	79% [20]	30 h [20]		2 L/kg [20]	
Ketoprofen	IV, IM	2.2 mg/kg q24 h	0.48 [5]		20–23 h [21]	4 h [21]	0.5 L/kg [21]	
Celecoxib	PO	2 mg/kg			13.6 [22]	4.5 h [22]	1.9 L/kg [22]	98.48 mL/h/kg [22]

**Table 2:** Route, recommended dose, IC_50_ COX 1:Cox 2 selectivity, bioavailability, elimination half life, time to maximum concentration (tmax), volume of distribution (Vd) and clearance of common NSAIDs used in equine practice in foals under one month of age.

Drug	Route	Recommended Dosage for Foals	Selectivity (IC_50_ COX 1:COX 2)	Bioavailability	Elimination Half Life (t½)	Time to Maximum Concentration in Plasma (Tmax)	Volume of Distribution (Vd)	Clearance
Phenylbutazone	PO, IV	4.4 mg/kg q12 h 1st day, 2.2 mg/kg q12 h subsequent days	0.302 [16]		7.4 h [23]		0.274 L/kg [20]	0.018 L/h/kg [20]
Flunixin	IV, PO	1.1 mg/kg q12 h or 0.25 mg/kg q8 h	0.336 [16]		8.5 h [14]			
Meloxicam	IV, PO	0.6 mg/kg q12 h	3.806 [16]	85–89%	2.48 h [24]	1.5 h [24]	0.12 L/kg [24]	154 mL/kg/h [24]
Firocoxib	IV, PO	0.1 mg/kg q24 h	268–643 [19]	65–75% [25]	11.04 h [20]	0.54 h [25]	1.8 L/kg [25]	96 mL/kg/h [25]
Ketoprofen	IV, IM	2.2 mg/kg q24 h	0.48 [5]		4.3 h [26]	4 h [26]	0.5 L/kg [26]	

## Data Availability

Not applicable.

## References

[1] Raja S.N., Carr D.B., Cohen M., Finnerup N.B., Flor H., Gibson S., Keefe F.J., Mogil J.S., Ringkamp M., Sluka K.A. (2020). The revised International Association for the Study of Pain definition of pain: Concepts, challenges, and compromises. Pain.

[2] Gleerup K.B., Lindegaard C. (2016). Recognition and quantification of pain in horses: A tutorial review. Equine Vet. Educ..

[3] Baller L.S., Hendrickson D.A. (2002). Management of equine orthopedic pain. Vet. Clin. N. Am.-Equine.

[4] Smith B.P., Van Metre D.C., Pusterla N. (2019). Large Animal Internal Medicine—E-Book.

[5] Knych H.K. (2017). Nonsteroidal Anti-inflammatory Drug Use in Horses. Vet. Clin. N. Am.-Equine.

[6] Davis J.L. (2017). Nonsteroidal anti-inflammatory drug associated right dorsal colitis in the horse. Equine Vet. Educ..

[7] Cook V.L., Blikslager A.T. (2015). The use of nonsteroidal anti-inflammatory drugs in critically ill horses. J. Vet. Emerg. Crit. Care.

[8] Lees P., Landoni M.F., Giraudel J., Toutain P.L. (2004). Pharmacodynamics and pharmacokinetics of nonsteroidal anti-inflammatory drugs in species of veterinary interest. J. Vet. Pharmacol. Ther..

[9] Cashman J.N. (1996). The Mechanisms of Action of NSAIDs in Analgesia. Drugs.

[10] Chambers J.P., Waterman A.E., Livingston A. (1995). The effects of opioid and α2adrenergic blockade on non-steroidal anti-inflammatory drug analgesia in sheep. J. Vet. Pharmacol. Ther..

[11] Kis B., Snipes J.A., Isse T., Nagy K., Busija D.W. (2003). Putative Cyclooxygenase-3 Expression in Rat Brain Cells. J. Cerebr. Blood F Met..

[12] Hernández-Avalos I., Valverde A., Ibancovichi-Camarillo J.A., Sánchez-Aparicio P., Recillas-Morales S., Osorio-Avalos J., Rodríguez-Velázquez D., Miranda-Cortés A.E. (2020). Clinical evaluation of postoperative analgesia, cardiorespiratory parameters and changes in liver and renal function tests of paracetamol compared to meloxicam and carprofen in dogs undergoing ovariohysterectomy. PLoS ONE.

[13] Chavez J.R., Ibancovichi J.A., Sanchez-Aparicio P., Acevedo-Arcique C.M., Moran-Muñoz R., Recillas-Morales S. (2015). Effect of Acetaminophen Alone and in Combination with Morphine and Tramadol on the Minimum Alveolar Concentration of Isoflurane in Rats. PLoS ONE.

[14] Davis J.L., Reed S.M., Bayly W.M., Sellon D.C. (2018). Chapter 2—Pharmacologic Principles. Equine Internal Medicine.

[15] Wallace J.L. (2008). Prostaglandins, NSAIDs, and Gastric Mucosal Protection: Why Doesn’t the Stomach Digest Itself?. Physiol. Rev..

[16] Beretta C., Garavaglia G., Cavalli M. (2005). COX-1 and COX-2 inhibition in horse blood by phenylbutazone, flunixin, carprofen and meloxicam: An in vitro analysis. Pharmacol. Res..

[17] Lees P., Toutain P.-L. (2013). Pharmacokinetics, pharmacodynamics, metabolism, toxicology and residues of phenylbutazone in humans and horses. Vet. J..

[18] Clark J.O., Clark T.P. (1999). Analgesia. Vet. Clin. N. Am.-Equine.

[19] Kvaternick V., Pollmeier M., Fischer J., Hanson P.D. (2007). Pharmacokinetics and metabolism of orally administered firocoxib, a novel second generation coxib, in horses. J. Vet. Pharmacol. Ther..

[20] Subhahar M. (2013). Pharmacokinetics and Pharmacodynamics of Some NSAIDs in Horses: A Pharmacological, Biochemical and Forensic Study. Ph.D. Thesis.

[21] Owens J.G., Kamerling S.G., Barker S.A. (1995). Pharmacokinetics of ketoprofen in healthy horses and horses with acute synovitis. J. Vet. Pharmacol. Ther..

[22] Subhahar M.B., Singh J., Albert P.H., Kadry A.M. (2019). Pharmacokinetics, metabolism and excretion of celecoxib, a selective cyclooxygenase-2 inhibitor, in horses. J. Vet. Pharmacol. Ther..

[23] Wilcke J.R., Crisman M.V., Sams R.A., Gerken D.F. (1993). Pharmacokinetics of phenylbutazone in neonatal foals. Am. J. Vet. Res..

[24] Raidal S.L., Edwards S., Pippia J., Boston R., Noble G.K. (2013). Pharmacokinetics and Safety of Oral Administration of Meloxicam to Foals. J. Vet. Intern. Med..

[25] Hovanessian N., Davis J.L., McKenzie H.C., Hodgson J.L., Hodgson D.R., Crisman M.V. (2014). Pharmacokinetics and safety of firocoxib after oral administration of repeated consecutive doses to neonatal foals. J. Vet. Pharmacol. Ther..

[26] Wilcke J.R., Crisman M.V., Scarratt W.K., Sams R.A. (1998). Pharmacokinetics of ketoprofen in healthy foals less than twenty-four hours old. Am. J. Vet. Res..

[27] Duz M., Marshall J.F., Parkin T.D. (2019). Proportion of nonsteroidal anti-inflammatory drug prescription in equine practice. Equine Vet. J..

[28] Marshall J.F., Blikslager A.T. (2011). The effect of nonsteroidal anti-inflammatory drugs on the equine intestine. Equine Vet. J..

[29] Noble G., Edwards S., Lievaart J., Pippia J., Boston R., Raidal S.L. (2012). Pharmacokinetics and Safety of Single and Multiple Oral Doses of Meloxicam in Adult Horses. J. Vet. Intern. Med..

[30] Walliser U., Fenner A., Mohren N., Keefe T., Devries F., Rundfeldt C. (2015). Evaluation of the efficacy of meloxicam for post-operative management of pain and inflammation in horses after orthopaedic surgery in a placebo controlled clinical field trial. BMC Vet. Res..

[31] Grauw J.C., Lest C.H.A., Brama P.A.J., Rambags B.P.B., Weeren P.R. (2009). In vivo effects of meloxicam on inflammatory mediators, MMP activity and cartilage biomarkers in equine joints with acute synovitis. Equine Vet. J..

[32] Raekallio M., Taylor P.M., Bennett R.C. (1997). Preliminary Investigations of Pain and Analgesia Assessment in Horses Administered Phenylbutazone or Placebo After Arthroscopic Surgery. Vet. Surg..

[33] Foreman J.H., Barange A., Lawrence L.M., Hungerford L.L. (2007). Effects of single-dose intravenous phenylbutazone on experimentally induced, reversible lameness in the horse. J. Vet. Pharmacol. Ther..

[34] Erkert R.S., MacAllister C.G., Payton M.E., Clarke C.R. (2005). Use of force plate analysis to compare the analgesic effects of intravenous administration of phenylbutazone and flunixin meglumine in horses with navicular syndrome. Am. J. Vet. Res..

[35] Owens J.G., Kamerling S.G., Stanton S.R., Keowen M.L., Prescott-Mathews J.S. (1996). Effects of pretreatment with ketoprofen and phenylbutazone on experimentally induced synovitis in horses. Am. J. Vet. Res..

[36] Doucet M.Y., Bertone A.L., Hendrickson D., Hughes F., Macallister C., McClure S., Reinemeyer C., Rossier Y., Sifferman R., Vrins A.A. (2008). Comparison of efficacy and safety of paste formulations of firocoxib and phenylbutazone in horses with naturally occurring osteoarthritis. J. Am. Vet. Med. Assoc..

[37] Orsini J.A., Ryan W.G., Carithers D.S., Boston R.C. (2012). Evaluation of oral administration of firocoxib for the management of musculoskeletal pain and lameness associated with osteoarthritis in horses. Am. J. Vet. Res..

[38] Hanson P.D., Brooks K.C., Case J., Conzemius M., Gordon W., Schuessler J., Shelley B., Sifferman R., Drag M., Alva R. (2006). Efficacy and safety of firocoxib in the management of canine osteoarthritis under field conditions. Vet. Ther..

[39] Moore J.N., Garner H.E., Shapland J.E., Hatfield D.G. (1981). Prevention of endotoxin-induced arterial hypoxaemia and lactic acidosis with flunixin meglumine in the conscious pony. Equine Vet. J..

[40] Werners A.H. (2017). Treatment of endotoxaemia and septicaemia in the equine patient. J. Vet. Pharmacol. Ther..

[41] Semrad S.D., Hardee G.E., Hardee M.M., Moore J.N. (1987). Low dose flunixin meglumine: Effects on eicosanoid production and clinical signs induced by experimental endotoxaemia in horses. Equine Vet. J..

[42] Barton M.H., Parviainen A., Norton N. (2004). Polymyxin B protects horses against induced endotoxaemia in vivo. Equine Vet. J..

[43] Tomlinson J.E., Blikslager A.T. (2005). Effects of cyclooxygenase inhibitors flunixin and deracoxib on permeability of ischaemic-injured equine jejunum. Equine Vet. J..

[44] Little D., Brown S.A., Campbell N.B., Moeser A.J., Davis J.L., Blikslager A.T. (2007). Effects of the cyclooxygenase inhibitor meloxicam on recovery of ischemia-injured equine jejunum. Am. J. Vet. Res..

[45] Urayama S., Tanaka A., Kusano K., Sato H., Nagashima T., Fukuda I., Fujisawa C., Matsuda H. (2019). Oral Administration of Meloxicam Suppresses Low-Dose Endotoxin Challenge–Induced Pain in Thoroughbred Horses. J. Equine Vet. Sci..

[46] Ziegler A.L., Freeman C.K., Fogle C.A., Burke M.J., Davis J.L., Cook V.L., Southwood L.L., Blikslager A.T. (2019). Multicentre, blinded, randomised clinical trial comparing the use of flunixin meglumine with firocoxib in horses with small intestinal strangulating obstruction. Equine Vet. J..

[47] Freeman D.E. (2019). Letter to the Editor: Multicentre, blinded, randomised clinical trial comparing the use of flunixin meglumine with firocoxib in horses with small intestinal strangulating obstruction. Equine Vet. J..

[48] Reisinger K.W., Schellekens D.H.S.M., Bosmans J.W.A.M., Boonen B., Hulsewé K.W.E., Sastrowijoto P., Derikx J.P.M., Grootjans J., Poeze M. (2017). Cyclooxygenase-2 Is Essential for Colorectal Anastomotic Healing. Ann. Surg..

[49] Ziegler A., Fogle C., Blikslager A. (2017). Update on the use of cyclooxygenase-2-selective nonsteroidal anti-inflammatory drugs in horses. J. Am. Vet. Med. Assoc..

[50] Bowen I.M., Redpath A., Dugdale A., Burford J.H., Lloyd D., Watson T., Hallowell G.D. (2020). BEVA primary care clinical guidelines: Analgesia. Equine Vet. J..

[51] Richardson L.M., Whitfield-Cargile C.M., Cohen N.D., Chamoun-Emanuelli A.M., Dockery H.J. (2018). Effect of selective versus nonselective cyclooxygenase inhibitors on gastric ulceration scores and intestinal inflammation in horses. Vet. Surg..

[52] Wang J., Qiu J., Xiao H., Gong X., Sun P., Li J., Zhang S., Cao X. (2020). Pharmacokinetics of three formulations of vitacoxib in horses. J. Vet. Pharmacol. Ther..

[53] Stewart A.J. (2020). Equine Analgesia for the Equine Practitioner.

[54] Reed S.K., Messer N.T., Tessman R.K., Keegan K.G. (2006). Effects of phenylbutazone alone or in combination with flunixin meglumine on blood protein concentrations in horses. Am. J.Vet. Res..

[55] Kivett L., Taintor J., Wright J. (2014). Evaluation of the safety of a combination of oral administration of phenylbutazone and firocoxib in horses. J. Vet. Pharmacol. Ther..

[56] Sanchez L.C., Reed S.M., Bayly W.M., Sellon D.C. (2018). Chapter 12—Disorders of the Gastrointestinal System. Equine Internal Medicine (Fourth Edition).

[57] Fletcher J.R., Bertin F.R., Owen H., Fraser N.S., Rose A.M., Stewart A.J. (2021). Oxytetracycline associated acute kidney injury in a neonatal foal. Equine Vet. Educ..

[58] Crisman M.V., Wilcke J.R., Sams R.A. (1996). Pharmacokinetics of flunixin meglumine in healthy foals less than twenty-four hours old. Am. J. Vet. Res..

[59] Semrad S.D., Sams R.A., Ashcraft S.M. (1993). Pharmacokinetics of and serum thromboxane suppression by flunixin meglumine in healthy foals during the first month of life. Am. J. Vet. Res..

[60] Carrick J.B., Papich M.G., Middleton D.M., Naylor J.M., Townsend H.G. (1989). Clinical and pathological effects of flunixin meglumine administration to neonatal foals. Can. J. Vet. Res..

[61] Traub-Dargatz J.L., Bertone J.J., Gould D.H., Wrigley R.H., Weiser M.G., Forney S.D. (1988). Chronic flunixin meglumine therapy in foals. Am. J. Vet. Res..

[62] Wilson K.E., Davis J.L., Crisman M.V., Kvaternick V., Zarabadipour C., Cheramie H., Hodgson D.R. (2017). Pharmacokinetics of firocoxib after intravenous administration of multiple consecutive doses in neonatal foals. J. Vet. Pharmacol. Ther..

[63] Pedersen S.K., Cribb A.E., Read E.K., French D., Banse H.E. (2018). Phenylbutazone induces equine glandular gastric disease without decreasing prostaglandin E2 concentrations. J. Vet. Pharmacol. Ther..

[64] Sykes B.W., Hewetson M., Hepburn R.J., Luthersson N., Tamzali Y. (2015). European College of Equine Internal Medicine Consensus Statement--Equine Gastric Ulcer Syndrome in Adult Horses. J. Vet. Intern. Med..

[65] Bjarnason I., Scarpignato C., Holmgren E., Olszewski M., Rainsford K.D., Lanas A. (2018). Mechanisms of Damage to the Gastrointestinal Tract From Nonsteroidal Anti-Inflammatory Drugs. Gastroenterology.

[66] Zushi S., Shinomura Y., Kiyohara T., Minami T., Sugimachi M., Higashimoto Y., Kanayama S., Matsuzawa Y. (1996). Role of prostaglandins in intestinal epithelial restitution stimulated by growth factors. Am. J. Physiol.-Gastrointest. Liver Physiol..

[67] Martínez Aranzales J., Cândido de Andrade B., Silveira Alves G. (2015). Orally administered phenylbutazone causes oxidative stress in the equine gastric mucosa. J. Vet. Pharmacol. Ther..

[68] Macallister C.G., Morgan S.J., Borne A.T., Pollet R.A. (1993). Comparison of Adverse-Effects of Phenylbutazone, Flunixin Meglumine, and Ketoprofen in Horses. J. Am. Vet. Med. Assoc..

[69] Meschter C.L., Gilbert M., Krook L., Maylin G., Corradino R. (1990). The effects of phenylbutazone on the morphology and prostaglandin concentrations of the pyloric mucosa of the equine stomach. Vet. Pathol..

[70] (2010). Expression of cyclooxygenase isoforms in ulcerated tissues of the nonglandular portion of the stomach in horses. Am. J. Vet. Res..

[71] D’Arcy-Moskwa E., Noble G.K., Weston L.A., Boston R., Raidal S.L. (2012). Effects of Meloxicam and Phenylbutazone on Equine Gastric Mucosal Permeability. J. Vet. Intern. Med..

[72] Wallace J.L., Syer S., Denou E., de Palma G., Vong L., McKnight W., Jury J., Bolla M., Bercik P., Collins S.M. (2011). Proton Pump Inhibitors Exacerbate NSAID-Induced Small Intestinal Injury by Inducing Dysbiosis. Gastroenterology.

[73] Gwee K.-A., Goh V., Lima G., Setia S. (2018). Coprescribing proton-pump inhibitors with nonsteroidal anti-inflammatory drugs: Risks versus benefits. J. Pain Res..

[74] Daniell H.W. (2012). NSAID–PPI Enteropathy in Humans. Gastroenterology.

[75] Jones S.M., Gaier A., Enomoto H., Ishii P., Pilla R., Price J., Suchodolski J., Steiner J.M., Papich M.G., Messenger K. (2020). The effect of combined carprofen and omeprazole administration on gastrointestinal permeability and inflammation in dogs. J. Vet. Intern. Med..

[76] Ricord M., Andrews F.M., Yñiguez F.J.M., Keowen M., Garza Jr F., Paul L., Chapman A., Banse H.E. (2021). Impact of concurrent treatment with omeprazole on phenylbutazone-induced equine gastric ulcer syndrome (EGUS). Equine Vet. J..

[77] Sulochana S.P., Syed M., Chandrasekar D.V., Mullangi R., Srinivas N.R. (2016). Clinical Drug–Drug Pharmacokinetic Interaction Potential of Sucralfate with Other Drugs: Review and Perspectives. Eur. J. Drug Metab. Pharmacokinet..

[78] Geor R.J., Petrie L., Papich M.G., Rousseaux C. (1989). The protective effects of sucralfate and ranitidine in foals experimentally intoxicated with phenylbutazone. Can. J. Vet. Res..

[79] Bishop R.C., Kemper A.M., Wilkins P.A., McCoy A.M. (2022). Effect of omeprazole and sucralfate on gastrointestinal injury in a fasting/NSAID model. Equine Vet. J..

[80] Read W.K. (1983). Renal Medullary Crest Necrosis Associated with Phenylbutazone Therapy in Horses. Vet. Pathol..

[81] Gunson D.E., Soma L.R. (1983). Renal Papillary Necrosis in Horses after Phenylbutazone and Water Deprivation. Vet. Pathol..

[82] Maxie M.G. (2007). Intestine. Jubb, Kennedy & Palmer’s Pathology of Domestic Animals.

[83] Karcher L.F., Dill S.G., Anderson W.I., King J.M. (1990). Right Dorsal Colitis. J. Vet. Intern. Med..

[84] McConnico R.S., Morgan T.W., Williams C.C., Hubert J.D., Moore R.M. (2008). Pathophysiologic effects of phenylbutazone on the right dorsal colon in horses. Am. J. Vet. Res..

[85] Cohen N.D. (2002). Right dorsal colitis. Equine Vet. Educ..

[86] Siwinska N., Zak A., Baron M., Cylna M., Borowicz H. (2017). Right dorsal colon ultrasonography in normal adult ponies and miniature horses. PLoS ONE.

[87] Jones S.L., Davis J., Rowlingson K. (2003). Ultrasonographic findings in horses with right dorsal colitis: Five cases (2000–2001). J. Am. Vet. Med. Assoc..

[88] Cargile J.L., Burrow J.A., Kim I., Cohen N.D., Merritt A.M. (2004). Effect of Dietary Corn Oil Supplementation on Equine Gastric Fluid Acid, Sodium, and Prostaglandin E2 Content before and during Pentagastrin Infusion. J. Vet. Intern. Med..

[89] McFadzean W.J.M., Love E.J. (2019). Perioperative pain management in horses. Equine Vet. Educ..

[90] Sanchez L.C., Robertson S.A. (2014). Pain control in horses: What do we really know?. Equine Vet. J..

[91] Mercer M.A., McKenzie H.C., Davis J.L., Wilson K.E., Hodgson D.R., Cecere T.E., McIntosh B.J. (2020). Pharmacokinetics and safety of repeated oral dosing of acetaminophen in adult horses. Equine Vet. J..

[92] Gordon E., Sandquist C., Cebra C.K., Heidel J., Poulsen K., Schlipf J.W. (2018). Esthesiometric evaluation of corneal analgesia after topical application of 1% morphine sulfate in normal horses. Vet. Ophthalmol..

